# Effects of multiple stress events at different stages of life on the incidence of metabolic syndrome

**DOI:** 10.3389/fendo.2024.1419443

**Published:** 2024-10-22

**Authors:** Na Li, Yuanyuan Gao, Xiaochuan Zhao, Lan Wang, Ran Wang, Mei Song, Peihua Hu, Wenting Lu, Tianyu Zhao, Fanfan Huang, Bufan Liu, Ruojia Ren, Xueyi Wang

**Affiliations:** ^1^ Department of Psychiatry, The First Hospital of Hebei Medical University, Shijiazhuang, China; ^2^ Mental Health Center, Hebei Medical University, Shijiazhuang, China

**Keywords:** multiple stress, metabolic syndrome, fetal, childhood, adulthood

## Abstract

**Objective:**

To investigate the effects of multiple stress events in different stages of life on the incidence of metabolic syndrome (MetS).

**Methods:**

Miners from Tangshan, China, were recruited for this study. Workers of the Kailuan Mining Group were evaluated to investigate whether exposure to Tangshan earthquakes during the fetal period in 1976. Adult life events and childhood trauma were assessed separately via the Life Event Scale and Childhood Trauma Questionnaire. The subjects were physically examined and general demographic data such as waist circumference were collected. Blood samples were collected for measurement of metabolic parameters. Corticotropin releasing hormone(CRH) levels was measured by enzyme-linked immunosorbent assay (ELISA). The subjects were divided into four groups according to their exposure to traumatic events in different stages of life: no exposure group, 1-exposure group, 2-exposures group, and 3-exposures group. The incidence of MetS, metabolic parameters and CRH levels in each of the four groups was compared.

**Results:**

In all, 626 people were enrolled; of these, 183, 262, 150, and 31 were in the no exposure, 1-exposure, 2-exposures, and 3-exposure groups, respectively. A remarkable variation in the incidence of MetS was observed among the four groups (*x^2^ =* 16.462, *P*<0.001). MetS incidence increased with the increasing number of traumatic events, except for in the no exposure group (17.9% in 1-exposure group, 24.7% in 2-exposure group, and 48.4% in the 3-exposure group). Multivariate logistic regression analysis showed that exposure to multiple stress during the fetal, childhood, and adult stages of life represent independent risk factors for developing MetS (*OR*=3.134, *95%CI*=1.042–9.429). Smoking increased the risk of developing MetS (*OR*=1.809, *95%CI*=1.140–2.871).

**Conclusions:**

Exposure to multiple traumatic events in distinct life stages increases the risk of developing MetS. Smoking is a risk factor for developing MetS.

## 1 Introduction

Metabolic syndrome (MetS) refers to a complex pathological group of metabolic disturbances. It includes hyperglycemia, hypertension, visceral obesity, and hyperlipidemia (elevated triglycerides, low high- density lipoproteins) as defined by the World Health Organization. Such factors constitute higher risks for developing type 2 diabetes, cardiovascular disease, and early mortality. Approximately 25% of the global population is estimated to be affected by MetS (1). MetS-related disturbances are the main causes of death globally, from non-communicable diseases (2). MetS prevalence among American adults is estimated to be approximately 34.7% (3). During 2011–2016, MetS prevalence among young people (20–39-year-olds) increased dramatically (from 16.2% to 21.3%) (3). In China, the prevalence of MetS is 33.9% (36.8% in female and 31.0% in male subjects), suggesting that MetS affects about 454 million adults (4). Additionally, the prevalence of MetS among younger people has doubled in China during 2002–2012 (5). In 2015–2017, more than 80% adults have at least one abnormal component of MetS (6). The younger the individual at MetS onset, the higher the risk of developing cardiovascular disease (7). These studies confirm that MetS is on the rise globally across all ages. Therefore, it is necessary to have an effective prevention and treatment strategy for controlling the MetS epidemic.

MetS is the result of a complex interaction between environmental factors and genetic factors and is influenced by the environmental effects occurring in early life. Numerous early-life events such as maternal conditions or stressors, adverse childhood environment, psychosocial stressors, and lifestyle and nutrition imbalance may lead to MetS (8, 9). The Dutch Famine Birth Cohort showed that children whose mothers experienced famine in pregnancy had a higher probability to be symptomatic for MetS, with respect to factors like abnormal blood lipid, adiposis, hypertension, and insulin resistance (10). In addition, we found from past earthquake stress research that perinatal exposure to earthquake stress increases the risk of developing MetS-related biomarkers (hypertension and diabetes) in adult life (11). However, previous studies of maternal stress during pregnancy and risk for developing MetS have ignored the effects arising from post-natal traumatic events such as adverse childhood experiences (ACEs) and adult life events.

ACE is a combination of various experiences such as neglect (physical and emotional) and all types of abuse (sexual, emotional, and physical). It may also include other unhappy matters that undermine children’ safety, bonding, and a sense of stability. ACE has been reported to have a long-term and far-reaching impact on mental health (12). It is closely related to physical health problems in general adult populations. However, existing research assessing the relationship of MetS with ACE is not consistent. A majority of the research studies have found a close relationship of ACE with at least one or more MetS components (13). Moreover, a strong epidemiological link between ACE and type 2 diabetes, hypertension, and dyslipidemia have been reported (14, 15). The incidence of abdominal obesity, as a core component of MetS, has increased in recent years. Large cohort studies have provided proof of a strong relation of adverse experiences in childhood with raised insulin resistance as well as body mass index (BMI) in later life (16, 17). However, some children who are exposed to ACE may not develop physical diseases. A recent study confirmed that childhood emotional abuse has significant positive relation with type 2 diabetes in adulthood (18). However, this study did not show a significant association between physical neglect, physical abuse, or sexual abuse and type 2 diabetes (18). Manandhar et al. failed to find an obvious relation of MetS in adulthood with parent’s absence in childhood (19).

There is increasing evidence to indicate that mental pressure in adulthood influences the physiological parameters of the human body including waist circumference and lipid profile. A cohort research on middle-to-old-age subjects found that persons suffering more Life Event Scale (LES) factors at baseline had a significantly increased waist circumference during the 6.5-year follow-up (20). Naharin et al. reported that the risk of hypertriglyceridemia in middle-aged men might be raised by stressful life events (21). However, none of these studies showed the relation of stressful life events with MetS as a whole. However, prior studies have focused on the impact of stressful life events on the risk of developing MetS during a limited period of follow-up, and did not consider the influence of the superimposed effects of stress on the incidence of MetS in later life.

Overall, these studies suggest that traumatic experiences can increase the risk of developing MetS. Hence, it seems likely that the propensity for developing MetS is not limited to single time-limited life stressors, rather it is likely modulated by the accumulation of multiple traumatic events throughout life. Here, we surveyed the incidence of MetS in people who suffered multiple traumatic events throughout life (fetal, childhood, and adult periods).

The HPA axis is a neuroendocrine system that regulates the stress response. In response to prolonged chronic stress, the hypothalamic paraventricular nucleus secretes corticotropin-releasing hormone (CRH). Extrahypothalamic CRH systems are the principal components of stress response (22). Elevated basal plasma cortisol levels are highly associated with type 2 diabetes and obesity (23, 24). It is unclear that repeated, multiple stresses throughout the human life span contribute to the increased risk of MetS by dysregulating the CRH system. Therefore, this study explored the mechanism of multiple stress and MetS by examining basal plasma CRH levels.

## 2 Subjects and methods

### 2.1 Subjects

Miners from Tangshan, China, were recruited for this study. All research participants were native-born Tangshan people living in the city since the date of birth. All subjects provided written consent for participation in the study. Three exposure factors occurring during different life stages were evaluated: earthquake exposure during fetal period, adverse childhood experiences, and adult life events. Participants whose mothers experienced the Tangshan earthquake on July 28, 1976, when pregnant with the participant were considered as having experienced prenatal trauma exposure. A 7.8-magnitude earthquake occurred on 28 July, 1976, in Tangshan, razing Tangshan city to the ground within a short duration. Tangshan suffered serious losses in power and water supply, transportation, and telecommunications, which led to 242,000 deaths and 164,000 serious injuries. The birth date of all participants considered to have experienced the earthquake was between 29 July, 1976 and 28 April, 1977. Participants born between July 29, 1977 and April 28, 1978 were not considered to have experienced the earthquake prenatally. The Childhood Trauma Questionnaire (CTQ) and Life Event Scale (LES) were adopted to assess traumatic events for all subjects both in childhood and adulthood. The Childhood Trauma Questionnaire Short Form (CTQ-SF) consists of 28 items and measures childhood abuse and neglect experiences that can be categorized as falling into five subtypes: emotional abuse (EA), physical abuse (PA), sexual abuse (SA), emotional neglect (EN) and physical neglect (PN) (25). We defined childhood trauma as having at least one of the following: EA≥13, PA≥10, SA≥8, EN≥15, or PN≥10 (26). We defined adulthood trauma as >32 points in the LES score (27, 28).

Subjects were divided into four groups according to the period of exposure they experienced:

1. no exposure group: prenatal earthquake stress (-) and childhood trauma (-) and adulthood trauma (-).
2. 1-exposure group:
  - prenatal earthquake stress (+) and childhood trauma (-) and adulthood trauma (-).
  - prenatal earthquake stress (-) and childhood trauma (+) and adulthood trauma (-).
  - prenatal earthquake stress (-) and childhood trauma (-) and adulthood trauma (+).
3. 2-exposure group:
  d. prenatal earthquake stress (+) and childhood trauma (+) and adulthood trauma (-).
  e. prenatal earthquake stress (+) and childhood trauma (-) and adulthood trauma (+).
  f. prenatal earthquake stress (-) and childhood trauma (+) and adulthood trauma (+).
4. 3-exposure group: prenatal earthquake stress (+) and childhood trauma (+) and adulthood trauma (+).

### 2.2 Subject evaluation

As per standardized protocol, trained interviewers gathered socio-demographic information; drinking & smoking history; illness history of diabetes mellitus, arterial hypertension, and hyperlipidemia; family history of diabetes mellitus and cardiovascular disease; as well as the participants’ mothers’ pregnancy situations during in-person interview. The interview considered different socio-demographic factors such as sex, age, monthly family income per capita (RMB) (≤2000, 2000–5000, and >5000); education background (≤6 years, 6–12 years, and >12 years); and marital status (married or unmarried). Participants were classified into three groups according to smoking status: never smoked, ex-smoker, and current smoker. Subjects were also divided into three groups as drinking status: current drinker, ex-drinker, and never drank.

### 2.3 Physical measurements

Corrected mercury sphygmomanometer was used to measure the blood pressure of the right brachial artery. Before blood pressure measurement, all participants avoided drinking coffee and tea as well as smoking, then sat still for 15 minutes. Blood pressure measurement was repeated three times with 1–2-min intervals. We averaged the obtained measurement outcomes. Waist circumference was evaluated by measuring body circumference midway between the iliac crest and lowest rib. Measurements were made parallel to the ground without compressing body tissue by using an inelastic tape measure.

### 2.4 Biochemical measurements

Blood samples were collected from fasting participants during 07:00 to 09:00 in the morning of the same day as the medical test. We drew blood from the cubital vein into EDTA vacuum tubes, centrifuged samples at 3000 g at room temperature for 10 min. Thereafter, we collected the supernatant to analyze the levels of fasting triglycerides (TG), fasting blood glucose (FBG), fasting serum total cholesterol triglycerides (TC), fasting high-density lipid cholesterol (HDL-C), and fasting low-density lipid cholesterol (LDL-C). TG and TC were measured by using the oxidase method (Shanghai Classical Biological Engineering Co., LTD.). The hexokinase method was used to measure FBG (Beikong Biotechnology Co.). All analysis was done using Hitachi 7600 Automatic Biochemical Analyzer, with quality control performed on each batch. CRH levels was measured by enzyme-linked immunosorbent assay (ELISA).

We defined MetS in line with guidelines for preventing and controlling type 2 diabetes in China (2017 Edition) (29). We diagnosed participants simultaneously having three or more symptoms as below with MetS: (1) abdominal adiposity: male waist circumference ≥ 90 cm or female waist circumference ≥ 85 cm. (2) HDL-C<1.04 mmol/L. (3) TG≥1.70 mmol/L. (4) FBG ≥ 6.1 mmol/L or blood glucose ≥7.8 mmol/L 2 h following glucose load and/or diagnosed with glycuresis and treated. (5) High blood pressure: diastolic blood pressure (DBP)≥85 mmHg or systolic blood pressure (SBP)≥130 mmHg or on medication for hypertension.

### 2.5 Statistical analyses

All statistical analysis performed with SPSS 23.0 (IBM Corporation, Armonk, NY, USA). The expression of measurement data was made using the mean and standard deviation. One-way ANOVA was used for comparison of continuous variables in all four groups, and chi-squared tests were used for comparison of categorical variables in all four groups. MetS influencing factors were identified via multivariate logistic regression analysis. Statistical significance was considered at a two-sided P-value of <0.05.

## 3 Results

### 3.1 Demographic features

**Figure 1:**
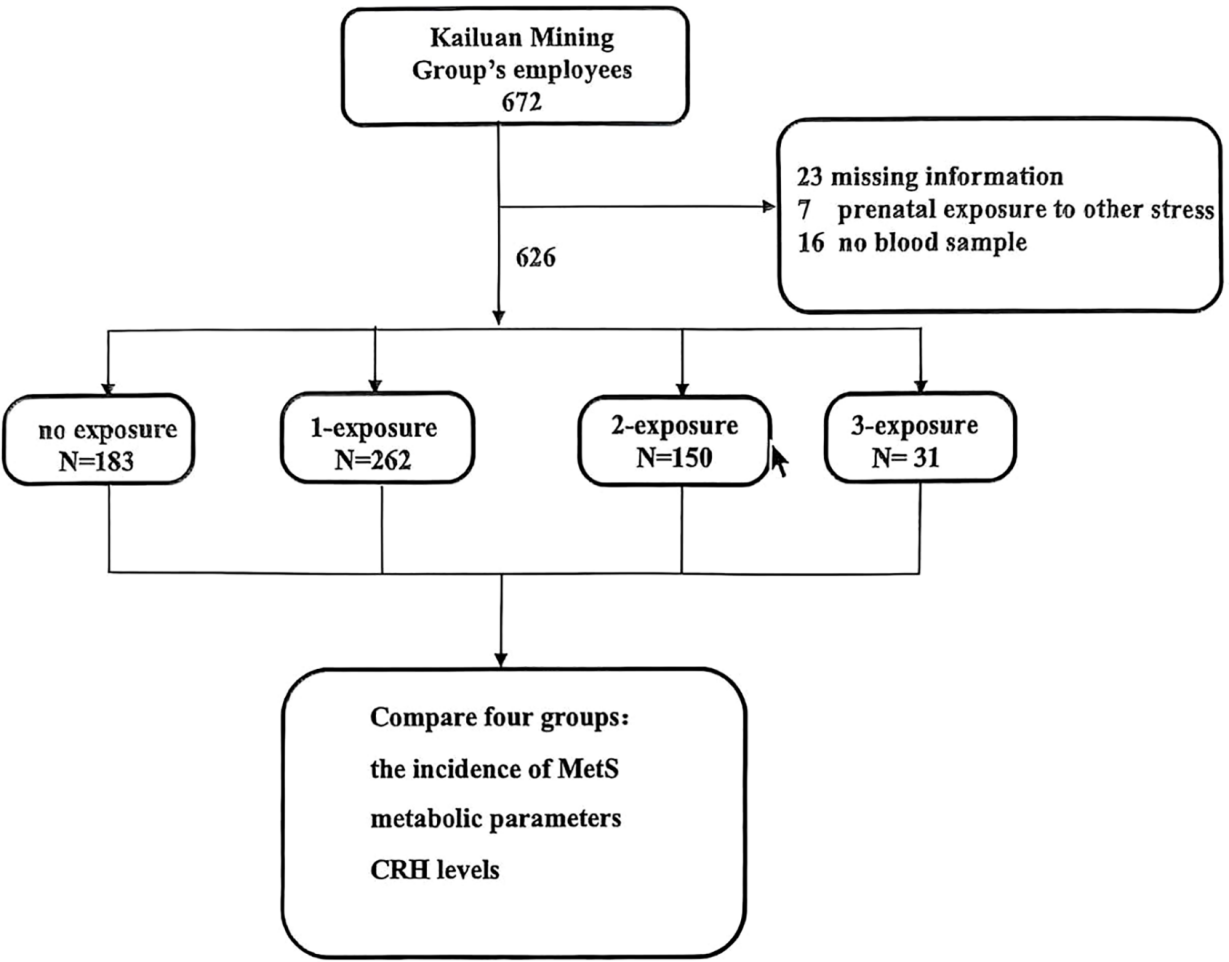
Enrollment, exclusion, and inclusion of Kailuan workers as study subjects. no exposure group: prenatal earthquake stress (-) and childhood trauma (-) and adulthood trauma (-); 1-exposure group: prenatal earthquake stress (+) and childhood trauma (-) and adulthood trauma (-); prenatal earthquake stress (-) and childhood trauma (+) and adulthood trauma (-); prenatal earthquake stress (-) and childhood trauma (-) and adulthood trauma (+); 2-exposure group: prenatal earthquake stress (+) and childhood trauma (+) and adulthood trauma (-); prenatal earthquake stress (+) and childhood trauma (-) and adulthood trauma (+); prenatal earthquake stress (-) and childhood trauma (+) and adulthood trauma (+); 3-exposure group: prenatal earthquake stress (+) and childhood trauma (+) and adulthood trauma (+).

We screened Kailuan Mining Group’s employees, and 672 employees agreed to participate in our study. Following questionnaire completion, we excluded 46 employees: 23 were excluded for missing information on earthquake exposure or other information; 7 were excluded because of prenatal exposure to other stress; and 16 for refusal to provide a blood sample for testing. The remaining 626 individuals were included in the analyses (**Figure 1**).

Of these subjects, 183 met the criteria for the no exposure group; 262 for the 1-exposure group; 150 for the 2-exposure group; and 31 for the 3-exposure group. Group disparities in the incidence of MetS were significant (*x^2^ =* 16.462, *P*<0.001) (**Table 1**). Except for the no exposure group (19.7%), the incidence of MetS increased with the increasing number of traumatic events: 17.9% in the 1-exposure group, 24.7% in the 2-exposure group, and 48.4% in the 3-exposure group. The MetS rate is significant different in the seven groups (*x^2^ =* 27.927, *P <*0.001) (**Table 2**). The incidence of MetS in 3-exposure group was significantly increased than no exposure group, prenatal earthquake stress group, childhood trauma group, childhood & adulthood trauma group (48.4% vs. 19.7%, 19.0%,14.3% and 11.8%) (**Table 2**). The proportion of MetS in prenatal earthquake stress & adulthood trauma group was significantly higher than childhood trauma group (39.2% vs. 14.3%) (**Table 2**).

**Table 1:** Characteristics of four groups.

	No	1-exposure	2-exposure	3-exposure	Test value	*P*
N	Exposure	group	group	group		
	183	262	150	31		
Gender, n (%)						
Male	168(91.8)	237(90.5)	145(96.7)	30(96.8)	7.254	0.064
Female	15(8.2)	25(9.5)	5(3.3)	1(3.2)		
Age	37.4 ± 0.6	37.5 ± 1.2	38.17 ± 1.0	38.5 ± 0.5	26.722	<0.001
Educational level, n (%)						
≤6 years	0(0)	3(1.1)	4(2.7)	2(6.5)	16.799	0.010
6~12 years	113(61.7)	179(68.3)	107(71.3)	23(74.2)		
>12 years	70(38.3)	80(30.5)	39(26.0)	6(19.4)		
Marital status						
Married	176(96.2)	237(90.5)	137(91.3)	28(90.3)	6.198	0.102
Unmarried	7(3.8)	25(9.5)	13(8.7)	3(9.7)		
Family income per month for every person (RMB)						
≤2000	38(20.8)	61(23.3)	38(25.3)	10(32.3)	7.501	0.277
2000~5000	134(73.2)	170(64.9)	99(66.0)	18(58.1)		
>5000	11(6.0)	31(11.8)	13(8.7)	3(9.7)		
Smoke, n (%)						
Never smoked	100(54.6)	131(50.0)	89(59.3)	15(48.4)	5.657	0.463
ex-smoker	16(8.7)	32(12.2)	12(8.0)	2(6.5)		
Current smoker	67(36.6)	99(37.8)	49(32.7)	14(45.2)		
Alchol use, n (%)						
Never-drank	48(26.2)	64(24.4)	28(18.7)	11(35.5)	5.447	0.488
ex- drinker	4(2.2)	8(3.1)	4(2.7)	1(3.2)		
Current drinker	131(71.6)	190(72.5)	118(78.7)	19(61.3)		
History of diabetes and cardiovascular disease						
No	132(72.1)	198(75.6)	119(79.3)	23(74.2)	2.334	0.506
Yes	51(27.9)	64(24.4)	31(20.7)	8(25.8)		
MetS						
No	147(80.3)	215(82.1)	113(75.3)	16(51.6)	16.462	<0.001
Yes	36(19.7)	47(17.9)	37(24.7)	15(48.4)		

**Table 2:** Comparison of the rate of MetS among seven groups.

Group		no exposure group	1-exposure group			2-exposure group			3-exposure groups	Test value	P
			a	b	c	d	e	f			
N		183	126	77	59	65	51	34	31		
MetS	, n (%)										
	No	147 (80.3) _a, b_	102 (81.0) _a, b_	66 (85.7) _b_	47 (79.7) _a, b, c_	52 (80.0) _a, b, c_	31 (60.8) _a, c_	30 (88.2) _a, b_	16 (51.6) _c_	27.927	<0.001
	Yes	36 (19.7) _a, b_	24 (19.0) _a, b_	11 (14.3) _b_	12 (20.3) _a, b, c_	13 (20.0) _a, b, c_	20 (39.2) _a, c_	4 (11.8) _a, b_	15 (48.4) _c_		

a= prenatal earthquake stress (+) and childhood trauma (-) and adulthood trauma (-); b= prenatal earthquake stress (-) and childhood trauma (+) and adulthood trauma (-); c= prenatal earthquake stress (-) and childhood trauma (-) and adulthood trauma (+); d= prenatal earthquake stress (+) and childhood trauma (+) and adulthood trauma (-); e= prenatal earthquake stress (+) and childhood trauma (-) and adulthood trauma (+); f= prenatal earthquake stress (-) and childhood trauma (+) and adulthood trauma (+).

### 3.2 Association of exposure to stressful events and CRH levels

**Figure 2:**
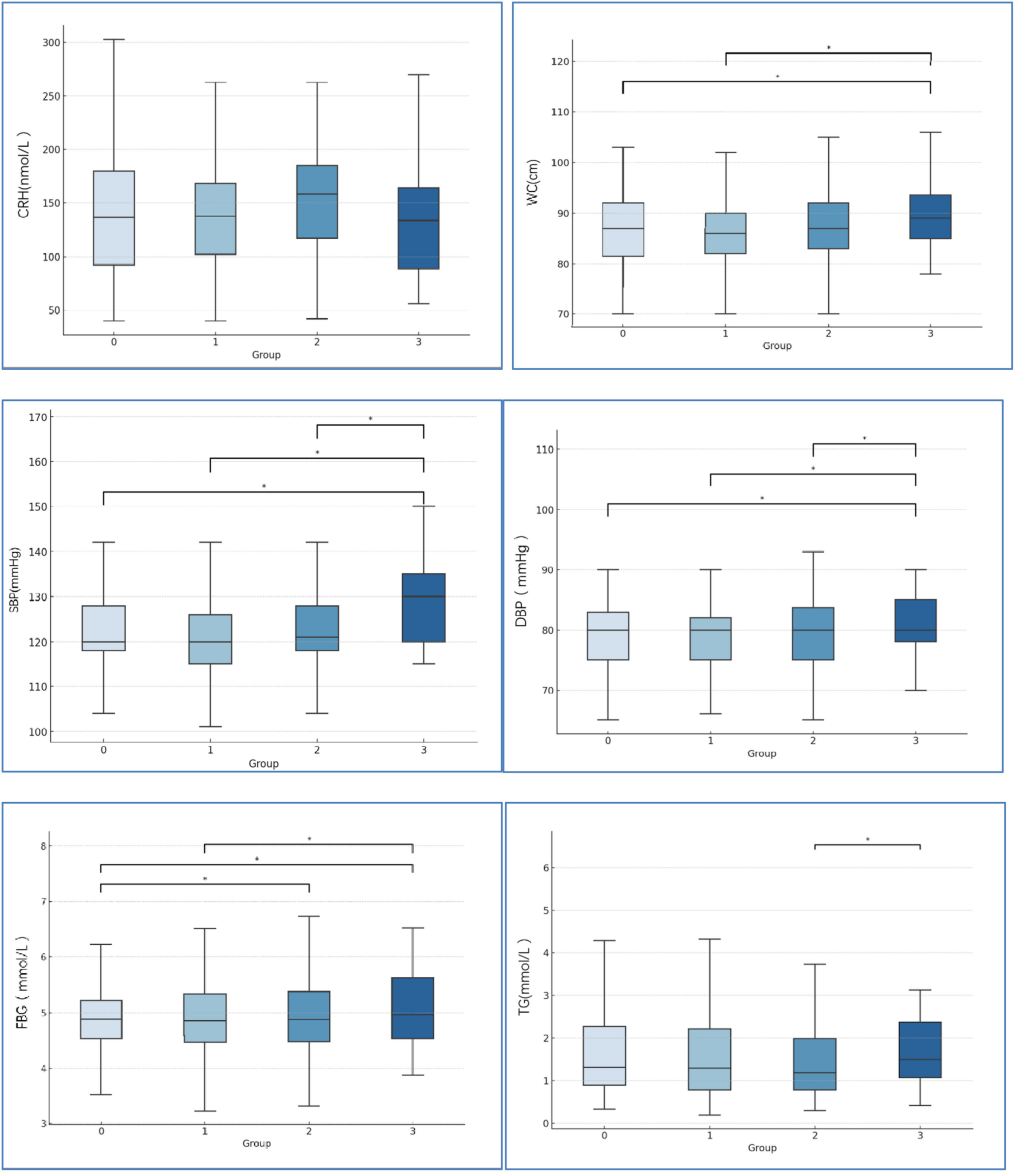
Comparison of CRH levels and the MetS-relate factors among four groups. 0=no exposure group, 1 = 1-exposure group, 2 = 2-exposure group, 3 = 3- exposure group. *P<0.05. CRH=corticotropin releasing hormone, WC, waist circumference; SBP, systolic blood pressure; DBP, diastolic blood pressure; FBG, fasting blood glucose; TG, fasting triglycerides.

No significant differences among the four groups were observed in CRH levels (**Figure 2**). For the mean of CRH levels of each group, no exposure group and 1-exposure group were similar (147.5 vs.143.5). 2-exposure group was mild higher than that in no exposure group (158.8 vs. 147.5). 3-exposure group was mild lower than that no exposure group (133.8 vs. 147.5).

### 3.3 Association of exposure to stressful events and the risk of MetS

Waist circumference, blood pressure, and metabolic parameters are shown in **Figure 2**. With increasing stress events, the mean of waist circumference, SBP, DBP, and FBG were significantly increased. No significant differences among the four groups were observed in HDL-C, and LDL-C.

### 3.4 Analysis of influencing factors of MetS

We adopted multivariate logistic regression analysis with/without MetS as the dependent variable to compare participant groups (**Table 3**). Education background, monthly family income, drinking and smoking status, stress exposure, and history of diabetes and cardiovascular disease were included as the independent variables. Multivariate logistic regression analysis found that exposure to multiple traumatic events during fetal, childhood, and adult life served as independent MetS risk factors (*OR*=3.134, *95%CI*=1.042–9.429). Smoking raised the risk of MetS (*OR*=1.809, *95%CI*=1.140–2.871). Other confounding factors did not enter the statistical equation.

**Table 3:** Multivariate logistic regression analysis of MetS risk factor.

	B	S.E.	Sig.	Exp(B)	95% CI	
No Exposure			0.001			
1 Exposure Factor	-0.038	0.529	0.943	0.963	0.342	2.715
2 Exposures Factor	0.071	0.526	0.892	1.074	0.383	3.012
3 Exposures Factor	1.142	0.562	0.042	3.134	1.042	9.429
Never-smoke			0.018			
ex-smoke	-0.164	0.453	0.718	0.849	0.35	2.062
Current-smoke	0.593	0.236	0.012	1.809	1.140	2.871
Constant	-1.829	0.525	0	0.161		

## 4 Discussion

In this study, workers who had been exposed to multiple stressful events during their fetal period, childhood, and adulthood were at significantly increased risk for developing MetS. Furthermore, the incidence of MetS increased with increasing number of traumas exposed at different stages of life. Numerous previous studies on animal models have reported that exposure to stress or corticosteroids during pregnancy can predict increased metabolic risk factors by changing the metabolic parameters (e.g., obesity, body mass, body fat, plasma leptin, SBP, plasma glucose, insulin, and blood lipid concentrations) (30, 31). Most animal studies have focused on glucocorticoid administration to rodents. Few similar studies have been performed in non-human primates. The Dutch Famine Birth Cohort Study discovered that children whose mothers experienced famine during pregnancy were more likely to suffer MetS symptoms such as dyslipidemia, insulin resistance, adiposis, and high blood pressure, suggesting an increased risk for developing MetS (10). Animal studies can readily control postnatal stress factors. However, it is difficult to control the effects of postnatal stress factors on psychosomatic diseases when studying human subjects. Previous researches about the risk of developing MetS have generally focused on the effects of stress during maternal pregnancy, but ignored the effects of re-exposure to stress during childhood and adulthood.

This study is consistent with prior research that suggested that cumulative ACEs are capable of increasing the risk of developing MetS. Jacqueline et al. showed that waist circumference and BMI steadily increase with increasing numbers (≥4) of ACEs (32). Delpierre et al. found that men experiencing two or more ACEs have a greater risk of developing MetS than those who are free from trauma exposure (33). However, certain stressors may represent a protective factor for MetS. Some research suggests that emotional neglect in childhood is a protective factor for type 2 diabetes in adulthood (18). Prior studies have focused on the association between traumatic events at one stage of life and MetS. Our study suggests that the risk for developing MetS is cumulative, and the risk increases as more stressors are encountered at later stages of life.

This study found that subjects who had experienced prenatal earthquake stress combined with adult stress had a significantly higher prevalence of MetS than those who had only experienced childhood trauma. However, there was no significant increase in the rate of MetS in the other 2-exposure group compared with the non-exposure group. This suggests that childhood trauma has a relatively mild impact on MetS risk in adulthood. This finding is relevant to population of our study: most participants were men. Pertinent studies to date have shown that childhood trauma is more strongly linked to mental illness than to somatic illnesses (34). For somatic diseases in general, stronger associations were observed in women than in men (34). Suglia and colleagues found that women who experienced sexual abuse in early childhood had a higher rate of hypertension, but among men, experiencing sexual abuse was not statistically significantly associated with hypertension (35). Maltreated females overall and neglected females in particular were at increased risk for above normal hemoglobin A1C (HbA1c) whereas the results for males were in the opposite direction (36). Elevated HbA1c levels indicating poor glycemic control and risk for diabetes. Future research should examine larger samples of female participants to compare gender differences.

Several researches have examined the relationship of multiple stressors with the risk of developing MetS. Recent research surveyed concomitant actions of multiple stressors on blood pressure reactivity. The investigators found that subjects exposed to severe childhood trauma or those who experienced persistent exposure to trauma had lower blood pressure reactivity and slower blood pressure recovery. Furthermore, joint actions of adversity in adulthood and childhood might have more powerful effects than adversity exposure only in childhood (37). This finding is consistent with our results. However, our study focuses on all symptoms of MetS and is not limited to blood pressure reactivity.

The study found that the CRH levels of subjects exposed to stress during two periods was mild higher than the no-exposed group, however subjects exposed to stress during all the three periods (pregnancy, childhood, and adulthood) had mild lower CRH Levels than no-exposed group. McEwen’s concept of allostatic overload may provide an explanation for the relation between CRH levels and stressful events (38). He suggested that harmful effects of stress on biological systems may arise while releasing multiple pressure mediators to support adaptation to stress. However, excessive, prolonged, and repeated over-release may lead to dysregulation and damage the body’s homeostasis (39). To maintain homoeostasis, hormones such as adrenaline, noradrenaline, and cortisol are released, which increases the activities of the peripheral and central nervous systems (CNS) to support adaptation to daily stressors. Cortisol regulates many basic biological systems such as immune function, metabolism, and inflammatory processes, whose function might be influenced by disruption of its circadian rhythm with long-term health implications. ACEs may be associated with cortisol response blunted to social stress later in life, and cumulative prolonged stressful events during early life may have peak expression in adulthood (40). Continued hypothalamic-pituitary-adrenal (HPA) axis maladaptation may result in stress disturbances like glycuresis, MetS, and high blood pressure (41).

In accordance with past research studies, this study found that current smoking status is a great risk for developing MetS. Prior research found that current smokers demonstrated greater insulin resistance than those who never smoked. Smoking cessation might prevent insulin resistance (42). A large South Korean research study evaluated drinking volume and MetS risk and found that ≤7.0 g/d alcohol volume reduced the risk of MetS development as compared to non-drinkers, but >7.0 g/d increased the risk (43). No relation of drinking volume with MetS was discovered in our study; this is perhaps because we did not differentiate between light and heavy drinkers.

## 5 Limitations and future research

Factors such as diet and exercise were not included in our analyses and may limit the generalizability of our findings. These factors were not included because of the difficulty of quantifying them. Moreover, the majority of subjects were men, hence limiting the generalizability of results to women. Nevertheless, there was no significant difference in the incidence of MetS between men and women in the four groups (**Table 1**). Future research should examine larger samples with greater numbers of female participants. It should also survey hormonal indicators relevant to stress in early life, such as the HPA axis-related hormones as well as biological markers associated with MetS including orexin, adiponectin, and ghrelin.

## Data Availability

The original contributions presented in the study are included in the article/supplementary material. Further inquiries can be directed to the corresponding author.
